# AUTS-2 Syndrome. Gravity comparison of three cases: a case series and review of the literature

**DOI:** 10.1192/j.eurpsy.2023.1880

**Published:** 2023-07-19

**Authors:** L. Delgado, C. Manso, A. Ruiz, C. Forcelledo, G. Fucho, M. Pàmies, D. J. Palao

**Affiliations:** 1 Mental Health; 2 Genetics, University Hospital Parc Taulí, Sabadell, Spain

## Abstract

**Introduction:**

Haploinssufficiency of AUTS2 gene has been associated with a syndromic form of neurodevelopmental delay called AUTS2 Syndrome (AUTS2S). It is characterized for having attention/hyperactivity disorder (ADHD), autism spectrum disorder (ASD), mild global development delay (GDD) and intellectual disability (ID). Clinicians also reported microcephaly, feeding difficulties, generalized hypotonia and ptosis.Due to its great variability, the AUTS2 Syndrome Severity Scoring System (ASSS) was established to assess the severity of the syndrome presentation. It is based on 32 characteristics including items of growth, feeding, neurodevelopment and congenital anomalies.

At the molecular level, the AUTS2 gene consists of 19 exons that are divided into a non-conserved N-terminal region and a conserved 3’ terminal end. There is a short isoform expressed primarily in the brain that initiates at an alternative transcription site and includes the last 11 exons. Variants that disrupt this final part of the gene have been associated with a severe phenotype.

**Objectives:**

To describe and compare 3 patients affected with AUTS2 syndrome using the ASSS.

**Methods:**

(1) Case series: Comparison of the patients diagnosed with AUTS2 Syndrome using the AUTS2 Syndrome Severity Score.

(2) Narrative review of the AUTS2 syndrome and the genotype-phenotype correlation through PubMed database (1990-2020). Key terms: “*AUTS2*”, “AUTS2 syndrome”, “ADHD”, “neurodevelopmental disorder”, “autism”.

**Results:**

1 (ASSS score: 12). Interstitial duplication long arm of chromosome 7. Characteristics: microcephaly, GDD, ASD features, ADHD, auditory hypersensitivity. Finger flexion and syndactylia, arched eyebrows, palpebral fissures, epicanthus, nares, micrognathia, narrow mouth.

2 (ASSS score: 13). Pathogenic variant exon 9. Characteristics: GDD, feeding problems, ID, ASD features, auditory hypersensitivity, ADHD, hypotonia, cerebral anomalies, hypertelorism, anteverted nostrils, broad nasal bridge, micrognathia, low-set ears, narrow mouth.

3 (ASSS score: 13). Pathogenic variant exon 16. Characteristics: ID, short stature, feeding problems, auditory hypersensitivity, ADHD, hypotonia, umbilical hernia, hypertelorism, proptosis, short palpebral fissures, epicanthus, prominent nasal tip, anteverted nares, low-set ears.

**Conclusions:**

Currently, 65 patients with pathogenic variants in AUTS2 are described in the literature. Significantly higher ASSS values have been found in patients with pathogenic variants affecting the 3’ end of the gene. Further research is needed, since genetic diagnosis of affected patients contributes to improved clinical protocols and personalized treatment.

**Disclosure of Interest:**

None Declared

